# Glycosylated Hazelnut Protein as Egg White Alternatives in Nougat Foams: Rheology, Texture, and Microstructure

**DOI:** 10.1002/fsn3.71955

**Published:** 2026-05-27

**Authors:** Ilyas Atalar, Abdullah Kurt, Sultan Demirci, Ceren Elmaci, Serdar Marasli, Nevzat Konar

**Affiliations:** ^1^ Food Engineering Department, Agriculture Faculty Eskisehir Osmangazi University Eskisehir Türkiye; ^2^ Food Engineering Department, Chemical and Metallurgical Engineering Faculty Yildiz Technical University Istanbul Türkiye; ^3^ ETI Food Research and Development Center Eskisehir Türkiye; ^4^ Agriculture Faculty, Dairy Technology Department Ankara University Ankara Türkiye; ^5^ Institute of Food Safety Ankara University Ankara Türkiye

**Keywords:** egg white replacer, glycosylation, hazelnut protein, nougat foam, rheology

## Abstract

This study examined Maillard‐type glycosylated hazelnut protein conjugates as plant‐based alternatives to egg white powder for producing aerated nougat foams. Hazelnut protein isolate (HPI) was conjugated with gum arabic (HPI–GA) or sodium alginate (HPI–SA) under optimized wet‐heating conditions and used to replace egg white powder at 25, 50, 75, and 100% of the control level. Foam performance was assessed by macroscopic appearance and apparent density, steady‐shear flow behavior (power‐law modeling), frequency‐dependent viscoelasticity (G′/G″), texture (spreadability and back extrusion), optical microstructure, and ATR–FTIR. Replacing egg white with native HPI weakened foam structure, yielding higher density, lower viscosity/consistency, and reduced mechanical resistance. In contrast, both conjugates improved foam structuring and deformation resistance, with HPI–SA providing the most pronounced reinforcement, particularly at 25%–50% replacement, where foam properties approached those of the egg white control while maintaining stable bubble dispersion. FTIR features supported the contribution of polysaccharide moieties and stronger hydration‐related interactions in conjugate systems. The findings indicate that glycosylated hazelnut proteins, especially HPI–SA, are promising functional substitutes for egg white in nougat foam formation.

## Graphical abstract

Maillard‐type glycosylation of hazelnut protein isolate with gum Arabic or sodium alginate improved the structural and functional properties of nougat foams. Glycosylated protein systems, particularly HPI–SA, enhanced foam stability, viscoelastic behavior, texture, and air bubble distribution, providing foam characteristics closer to those of egg white–based systems. These findings highlight the potential of glycosylated hazelnut proteins as plant‐based alternatives to egg white in aerated confectionery products.

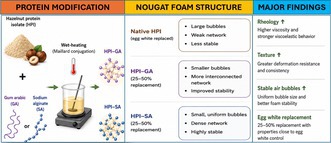

## 1 Introduction

Protein foams constitute the fundamental structural elements of many aerated food products, including cakes, breads, ice cream, and confectionery systems such as nougat fillings, where the incorporation and retention of air play a decisive role in determining texture and product quality (Davis et al. 2004; Foegeding et al. 2006). In such systems, the stability and functionality of the foam matrix are critical, as the original foam structure must withstand mechanical stresses during processing while maintaining sufficient aeration (Friberg et al. 2003). However, protein‐based foams are inherently unstable, which often complicates their use under industrial processing conditions.

Among food proteins, egg white proteins have long been regarded as benchmark foaming agents owing to their strong surface activity, rapid adsorption at the air–water interface, and ability to form stable aerated structures in products such as meringues, baked goods, and aerated confectionery systems (Bonilla et al. 2022). For this reason, egg white foams are often used as model systems to study protein‐driven foam formation and stabilization. The high amphiphilicity of egg white proteins allows them to rearrange quickly and adsorb at the air–water interface during whipping, with hydrophilic groups orienting toward the aqueous phase and hydrophobic groups toward the air phase. This behavior helps surround air bubbles and maintain foam stability (Dullien 2012; Liu et al. 2022; Narsimhan and Xiang 2018). The unique combination of rapid interfacial adsorption, cohesive film formation, and resistance to deformation allows egg white foams to maintain structural integrity during processing and setting (Chesterton et al. 2015; Lomakina and Mikova 2006). As a result, few alternative protein systems have been able to match the foaming performance of egg white in demanding foam‐based applications.

Despite these advantages, growing concerns related to allergenicity, animal welfare, sustainability, and cost volatility have intensified interest in identifying alternative protein sources capable of delivering comparable foaming functionality (Asghari et al. 2016). This has driven increasing research efforts toward plant‐based proteins and protein modification strategies aimed at improving foam stability, structure, and rheological performance in aerated food systems.

In recent years, increasing attention has been directed toward the development of plant‐based alternatives to egg proteins in food systems where foaming and rheological performance are critical. Several studies have demonstrated that plant protein conjugation can effectively enhance functional and structural properties relevant to egg white replacement. For example, guar gum–conjugated pea protein exhibited improved emulsifying performance, higher apparent viscosity, and enhanced viscoelastic behavior compared to native pea protein, highlighting the role of polysaccharide conjugation in reinforcing structural integrity (Y. Shen et al. 2022). Similarly, the use of a tri‐component hydrocolloid system composed of gellan gum, soy protein isolate, and maltodextrin as an egg white replacer in meringues resulted in increased foaming capacity and higher storage and loss moduli (G′ and G″), indicating improved rheological stability and foam structure (Choi et al. 2024). Beyond polysaccharides, covalent conjugation of soy protein with polyphenols has also been shown to improve solubility, emulsification, foaming, and gelling behavior, further supporting the potential of protein modification strategies for enhancing the functionality of plant‐based egg replacements (Qiu et al. 2025). Collectively, these findings demonstrate that protein conjugation represents a promising approach for improving the rheological and foaming performance of plant proteins, thereby supporting their use as functional alternatives to egg white in aerated food systems.

Recent research has increasingly focused on the interactions between proteins and polysaccharides in real food systems, as controlling these interactions is essential for tailoring the functional properties of food matrices (Asghari et al. 2016). The presence of polysaccharides can modify protein functionality, including surface activity, conformational stability, and foaming performance, by altering the structure and organization of the adsorbed interfacial layer (Asghari et al. 2016; Damodaran 1997; Schmidt et al. 2010). These interactions may be associative or dissociative in nature and can arise from a combination of electrostatic attractions, hydrogen bonding, and other short‐range forces, depending on the molecular characteristics of the components involved (Dickinson 2010; Patino and Pilosof 2011). In this context, Maillard‐type protein–polysaccharide conjugation has emerged as an effective modification strategy, enabling the covalent attachment of polysaccharide moieties to proteins and thereby enhancing interfacial stability, hydration capacity, and resistance to structural collapse under processing conditions. Such conjugation‐driven modifications offer a promising route to improve protein foaming performance in aerated food systems.

In previous studies, Maillard‐type conjugation of hazelnut protein isolate with sodium alginate and gum arabic was shown to markedly improve solubility, interfacial activity, and rheological behavior compared to the native protein. These improvements were associated with conformational rearrangements, increased molecular flexibility, and enhanced exposure of functional groups, which collectively strengthened protein–interface interactions (Atalar et al. 2025; Atalar et al. 2026). Such conjugation‐induced modifications provide a strong mechanistic basis for using glycosylated hazelnut proteins as functional alternatives to egg white in aerated food systems.

Although foaming capacity and stability are commonly evaluated in plant protein conjugate systems, their performance in complex real food applications, particularly aerated confectionery matrices, remains insufficiently explored. Despite the well‐established use of protein–polysaccharide conjugation to improve foaming properties, studies focusing on underexplored plant protein sources in such systems are still scarce. In particular, the application of hazelnut protein in conjugated form for foam‐based confectionery products such as nougat has not been systematically investigated.

Therefore, this study investigates the potential of glycosylated hazelnut protein conjugates as functional alternatives to egg white proteins during the foam formation stage of nougat filling production. Nougat fillings are typically produced via a two‐step process, in which a foam‐like intermediate is first generated before incorporation into the fat–cocoa phase, making the stability and functionality of this initial foam phase critically important. Accordingly, hazelnut protein isolate conjugated with gum arabic or sodium alginate was evaluated in terms of macroscopic appearance, apparent density, textural properties, rheological behavior, and microstructural characteristics.

## 2 Material and Methods

### 2.1 Materials

Blanched hazelnut ( *Corylus avellana* ) samples of the Tombul variety were obtained from Yavuzkan Hazel Gıda (Giresun, Turkey). Hazelnut oil was separated by a cold‐pressing method, and hazelnut protein isolate (HPI) was subsequently obtained. The composition of HPI was determined as 97.05% dry matter, 80.15% protein, 10.15% fat, 5.85% carbohydrate, and 1.60% ash. Wet‐heat glycosylation of HPI with sodium alginate (SA) and gum Arabic (GA) was performed using a protein–saccharide ratio of 6% (w/w). The optimum conjugation conditions were 65°C for 5 h with a protein content of 35% for the HPI–SA system, and 71.5°C for 6.25 h with a protein content of 46% for the HPI–GA system. The optimum conditions were determined by simultaneously considering minimum hydroxymethylfurfural (HMF) formation and maximum degree of glycation, solubility, and emulsifying capacity. Sodium alginate and gum Arabic were purchased from Sigma‐Aldrich (St. Louis, USA). The following ingredients were supplied by European Union–based companies: egg white powder, glucose syrup and crystalline sugar (sucrose).

### 2.2 Nougat Foam Preparation

Nougat foam was prepared using an industrially relevant aeration process designed to generate a foam‐like sugar matrix. A sugar syrup was first produced by heating a mixture of water, sucrose, and glucose syrup until complete dissolution. After cooling to the target processing temperature, aeration was achieved by incorporating the protein‐based structuring agent under controlled mixing conditions to form a stable foam structure. The control formulation contained egg white powder (0.47%, w/w) as the conventional foaming agent. In experimental formulations, egg white powder was partially or fully replaced by either non‐glycosylated hazelnut protein isolate (HPI) or glycosylated hazelnut protein conjugates (HPI–GA or HPI–SA) at replacement levels of 25%, 50%, 75%, and 100% relative to the egg white powder content of the control formulation (0.47%, w/w), corresponding to protein or conjugate addition levels of 0.1175%–0.4700% (w/w). All nougat foam samples were prepared under identical processing conditions and were characterized in terms of macroscopic appearance, apparent density, rheological properties, and textural characteristics.

### 2.3 Density

Apparent density of the nougat foam samples was determined using a gravimetric method based on the mass‐to‐volume ratio. Foam samples were gently transferred into a container of known volume without compression, weighed, and apparent density (g/mL) was calculated as the ratio of sample mass to container volume (El‐Sayed and Hashim 2024).

### 2.4 Rheological Analyses

The rheological properties of the nougat foams were determined using a rotational rheometer (HAAKE Mars III, Thermo Scientific, Germany) equipped with a cone‐and‐plate geometry (diameter: 35 mm; cone angle: 2°; gap: 0.150 mm). All measurements were performed in triplicate (Atalar et al. 2021).

For steady‐shear flow behavior analysis, the samples were subjected to a continuous shear rate sweep ranging from 0 to 120 s^−1^ over a period of 2 min at 20°C. The flow behavior was described using the Ostwald–de Waele (power‐law) model, as expressed by the following equation (Equation 1)

(1) $$\tau =K{\dot{\gamma }}^{n}$$

where *τ* is the shear stress (Pa), $\dot{\gamma }$ is the shear rate (s^−1^), *K* is the consistency coefficient (Pa.s^n^), and *n* is the flow behavior index (dimensionless).

Frequency sweep measurements were performed within the linear viscoelastic region (LVR) at 20°C over an angular frequency range of 1–100 rad/s at a constant stress of 1 Pa, in order to evaluate the storage (G′) and loss (G″) moduli.

### 2.5 Texture Analysis

Spreadability and back extrusion measurement were performed using the Texture analyzer (TA XT. Plus, Stable Micro System, Surrey, UK). These experiments were carried out in five repetitions.

#### 2.5.1 Spreadability

The nougat foam samples were loaded into the TTC Spreadability Rig (HDP/SR) at room temperature. The analysis settings were as follows: a probe pretest distance of 55 mm, a test speed of 3 mm s^−1^ and a compression distance of 23 mm. The firmness and shear properties of the samples were determined by the device software (Texture Exponent Version 6.1.16.0) (Aydemir and Atalar 2019).

#### 2.5.2 Back Extrusion

The back‐extrusion test was performed in a reverse extrusion container (50 mm diameter) containing 75% of the sample using A/BE‐40 extrusion rig. The parameters used were as follows: the pre‐test and test speeds were set to 1 mm/s, the post‐test speed to 10 mm/s, the distance to 15 mm, and the triggering force to 10 g (Espert et al. 2019).

### 2.6 Microstructural Imaging of Nougat Foams

Microstructural observations of the nougat foam samples were performed using an optical light microscope equipped with a digital camera. Fresh foam samples were carefully placed on a glass slide to minimize structural disruption and covered with a coverslip without applying excessive pressure. Images were acquired under bright‐field illumination at the same magnification and optical settings for all samples to allow reliable qualitative comparison of foam microstructure. Representative micrographs were recorded from at least three randomly selected fields of view per sample (Liang et al. 2025) and all observations were conducted on independently prepared samples to ensure reproducibility.

### 2.7 Attenuated Total Reflection‐Fourier Transform Infrared (ATR‐FTIR)

The molecular structure of nougat foam samples was analyzed using ATR‐FTIR (Bruker Alpha II, Ettlingen, Germany). Samples were placed directly onto the ATR crystal, ensuring complete surface coverage. Spectra were recorded over the wavenumber range of 600–4000 cm^−1^ at a resolution of 4 cm^−1^ with 27 scans. Background spectra were recorded under identical conditions prior to each measurement and automatically subtracted from the sample spectra. Baseline correction and smoothing were applied to all spectra. The resulting data were analyzed using Origin 8.5.1 software to identify the main absorption bands (Aydemir et al. 2025).

### 2.8 Statistical Analysis

One‐way analysis of variance (ANOVA) with SPSS software (version 18.0, SPSS Inc., Chicago, USA) was employed to analyze the data, and the comparison of means was made using Tukey's test at a significance level of *p* < 0.05.

## 3 Results and Discussion

### 3.1 Macrostructural Appearance and Apparent Density of Nougat Foam

**FIGURE 1:**
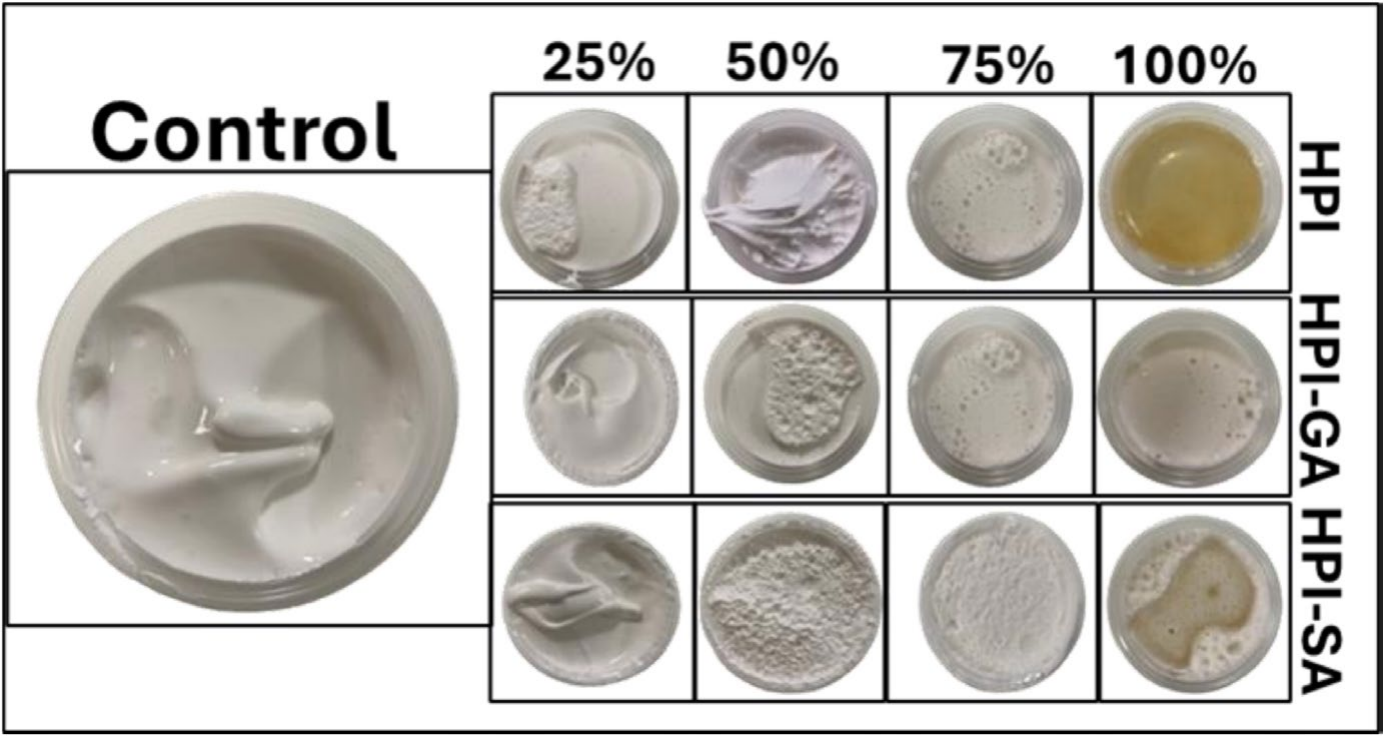
Macroscopic appearance of nougat foam samples with partial or complete egg white replacement using HPI, HPI–GA, and HPI–SA (25%–100%). The control sample is shown on the left. Samples prepared with HPI, HPI–GA, and HPI–SA are presented in rows, while columns represent increasing replacement levels (25%, 50%, 75%, and 100%).

The macroscopic appearance of the nougat foam samples is shown in Figure 1. The control formulation exhibited a smooth, cream‐gray, and homogeneous surface with no visible macroscopic air voids, indicating effective foam formation. Replacing egg white powder with alternative protein systems led to noticeable changes in foam appearance. Foams prepared with native HPI displayed a less uniform structure, suggesting a limited ability to stabilize the aerated sugar matrix. In contrast, foams containing glycosylated protein systems showed improved macroscopic characteristics. At a 25% replacement level, HPI–GA‐ and HPI–SA‐based foams most closely resembled the control, exhibiting relatively smooth and homogeneous structures, although they appeared slightly more fluid. As the replacement level increased to 50% and above, the foam structure gradually became rougher and more porous. At higher protein or conjugate contents (50%–100%), excessive structuring and loss of uniformity were evident, indicating reduced aeration efficiency.

These visual observations were supported by the apparent density results presented in Table 1. The control sample showed the lowest apparent density (0.54 g/mL), reflecting efficient air incorporation and a well‐aerated foam structure. Partial or complete replacement of egg white powder resulted in a progressive increase in apparent density for all protein systems, indicating reduced aeration efficiency and increased structural compactness. While apparent density increased with replacement level in all cases, the rate of densification differed markedly among formulations. Native HPI exhibited the steepest increase in apparent density with increasing replacement level, whereas HPI–GA and HPI–SA showed a more gradual increase up to 50% replacement, followed by a sharper rise at higher levels. At high replacement levels (75%–100%), all foams exhibited significantly higher density values, consistent with excessive structuring and limited air retention. Among the tested systems, HPI–SA at 25% and 50% replacement levels showed density values closest to the control, followed by HPI–GA at the same levels.

**TABLE 1:** Apparent density and textural properties of nougat foam samples.

Sample	Apparent density (g/mL)	Spreadability		Back extrusion	
		Firmness (g)	Work of shear (gs)	Peak force (g)	Positive area (gs)
**Control**	0.54 ± 0.02ᵃ	1820.17 ± 132.12ᵇ	1284.20 ± 122.30ᶜ	1145.00 ± 45.00ᶜ	28475.80 ± 399.80ᵈᵉᶠ
**25% HPI**	0.68 ± 0.03ᵇᶜ	1016.69 ± 42.00ᶠ	699.84 ± 7.47ᶠ	755.20 ± 5.20ᶠ	18845.30 ± 125.30ᶜᵈᵉ
**50% HPI**	0.79 ± 0.03ᶜᵈ	1454.17 ± 6.90ᵈ	1011.88 ± 44.67ᵈ	1036.45 ± 11.45ᵈ	25694.85 ± 285.85ᵇᶜᵈ
**75% HPI**	0.88 ± 0.04ᵈ	1136.62 ± 73.29ᵉᶠ	804.71 ± 45.11ᵉᶠ	644.70 ± 19.00ᵍ	17077.45 ± 517.45ᶜᵈᵉ
**100% HPI**	1.34 ± 0.05ᶠ	83.93 ± 0.99ᵍ	43.29 ± 2.95ʰ	71.45 ± 1.45ʲ	1402.30 ± 9.30ᶠ
**25% HPI–GA**	0.68 ± 0.03ᵇᶜ	1276.04 ± 65.15ᵉ	931.03 ± 54.26ᵈᵉ	908.65 ± 1.35ᵉ	24524.70 ± 1155.30ᵇᶜᵈ
**50% HPI–GA**	0.70 ± 0.03ᵇᶜ	1533.23 ± 43.87ᶜᵈ	1063.71 ± 25.40ᵈ	1119.65 ± 19.65ᶜ	29913.55 ± 521.55ᵇᶜ
**75% HPI–GA**	1.02 ± 0.04ᵉ	1496.72 ± 61.01ᶜᵈ	935.45 ± 26.10ᵈᵉ	1015.30 ± 4.70ᵈ	25720.65 ± 126.35ᵇᶜᵈ
**100% HPI–GA**	1.06 ± 0.04ᵉ	997.73 ± 7.58ᶠ	541.62 ± 40.94ᵍ	496.95 ± 3.05ʰ	13443.55 ± 82.45ᵈᵉᶠ
**25% HPI–SA**	0.60 ± 0.02ᵃᵇ	1851.45 ± 57.25ᵇ	1416.91 ± 12.25ᵇ	1364.50 ± 35.50ᵇ	32910.00 ± 856.00ᵇ
**50% HPI–SA**	0.63 ± 0.03ᵃᵇ	2564.67 ± 51.59ᵃ	1954.23 ± 87.62ᵃ	2087.85 ± 2.85ᵃ	50602.45 ± 61.45ᵃ
**75% HPI–SA**	0.80 ± 0.03ᶜᵈ	1613.19 ± 30.19ᶜ	1068.30 ± 13.06ᵈ	1011.55 ± 11.55ᵈ	26778.95 ± 304.95ᵇᶜᵈ
**100% HPI–SA**	1.36 ± 0.05ᶠ	1078.01 ± 25.40ᶠ	982.18 ± 16.49ᵈ	291.95 ± 8.05ᶦ	7748.80 ± 225.20ᵉᶠ

*Note:* Values are expressed as mean ± standard deviation (*n* = 3). Different superscript letters within the same column indicate significant differences (*p* < 0.05).

This behavior suggests that sodium alginate contributed to improved foam structure and air retention, likely because of its higher solubility and stronger interactions at the air–water interface compared to native HPI. In addition, its viscous nature may have promoted the formation of a more stable interfacial layer, reducing moisture loss and enhancing foam stability (Wang et al. 2020). In contrast, foams prepared with native HPI showed a pronounced increase in apparent density even at moderate replacement levels, reflecting a lower foam‐stabilizing efficiency. These trends are consistent with the rougher and more compact macroscopic appearance observed in Figure 1. Overall, the observed differences in apparent density can be linked to variations in foamability, which depend on protein adsorption at the air–water interface, resistance of the interfacial layer to shear during whipping, and the ability of the interfacial film to maintain bubble stability after formation (P. Shen et al. 2024).

### 3.2 Rheological Behaviors of Nougat Foam

#### 3.2.1 Steady Shear

**FIGURE 2:**
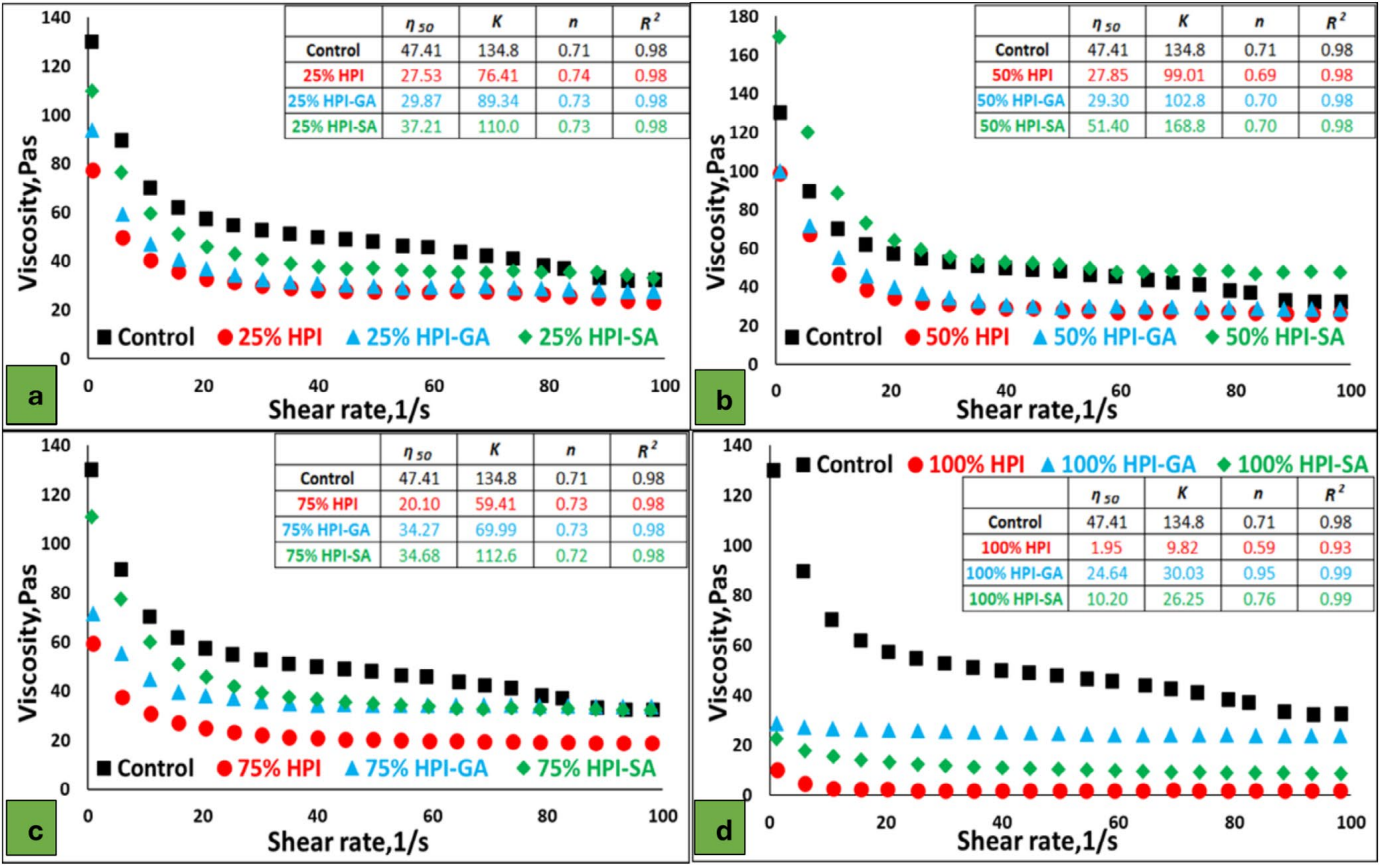
Steady‐shear viscosity profiles and corresponding power‐law parameters of nougat foam samples at different replacement levels: (a) 25%, (b) 50%, (c) 75%, and (d) 100%. Control and samples formulated with HPI, HPI–GA, and HPI–SA are presented in each panel. Insets show the corresponding power‐law (Ostwald–de Waele) model parameters, including apparent viscosity at 20 s^−1^ (η50), consistency index (*K*), flow behavior index (*n*), and coefficient of determination (*R* ^2^).

The steady‐shear flow behavior of the nougat foam samples is presented in Figure 2. All formulations exhibited pronounced shear‐thinning behavior, characterized by a decrease in apparent viscosity with increasing shear rate, which is typical of aerated and structured food foams. The flow curves were well described by the Ostwald–de Waele model, with high coefficients of determination (R^2^ ≥ 0.98), confirming the suitability of the power‐law approach for these systems.

Compared to the control, replacing egg white powder with alternative protein systems led to clear differences in viscosity profiles. At low replacement levels (25% and 50%), foams prepared with glycosylated proteins (HPI–GA and HPI–SA) exhibited viscosity values close to those of the control over the entire shear rate range. In contrast, foams containing native HPI showed lower viscosity, particularly at low shear rates, indicating the formation of a weaker structural network.

The higher apparent viscosity observed for the conjugate‐containing systems can be attributed to several concurrent mechanisms, including increased steric hindrance due to molecular expansion, enhanced water binding associated with hydrated saccharide groups, and, most importantly, the formation of protein‐based polymers with higher molecular weight and larger hydrodynamic volume (Bönisch et al. 2007; Song and Zhao 2013).

As the replacement level increased to 75% and 100%, differences among formulations became more pronounced. Foams prepared with native HPI showed a substantial reduction in viscosity and lower consistency index (K), reflecting limited resistance to shear and reduced structural integrity. In contrast, glycosylated protein systems (particularly HPI–SA) maintained higher viscosity and consistency values, indicating improved network formation and greater resistance to deformation under shear (Lazidis et al. 2016). For all samples, the flow behavior index (*n*) values remained below unity, confirming shear‐thinning behavior. However, glycosylated protein systems exhibited slightly higher n values than native HPI, suggesting a more stable shear response and enhanced structural resilience of the foam network. These rheological results demonstrate that protein glycosylation, especially with sodium alginate, significantly improved the steady‐shear rheological performance of nougat foams, in agreement with the trends observed in apparent density and macroscopic appearance.

#### 3.2.2 Dynamic Viscoelastic

**FIGURE 3:**
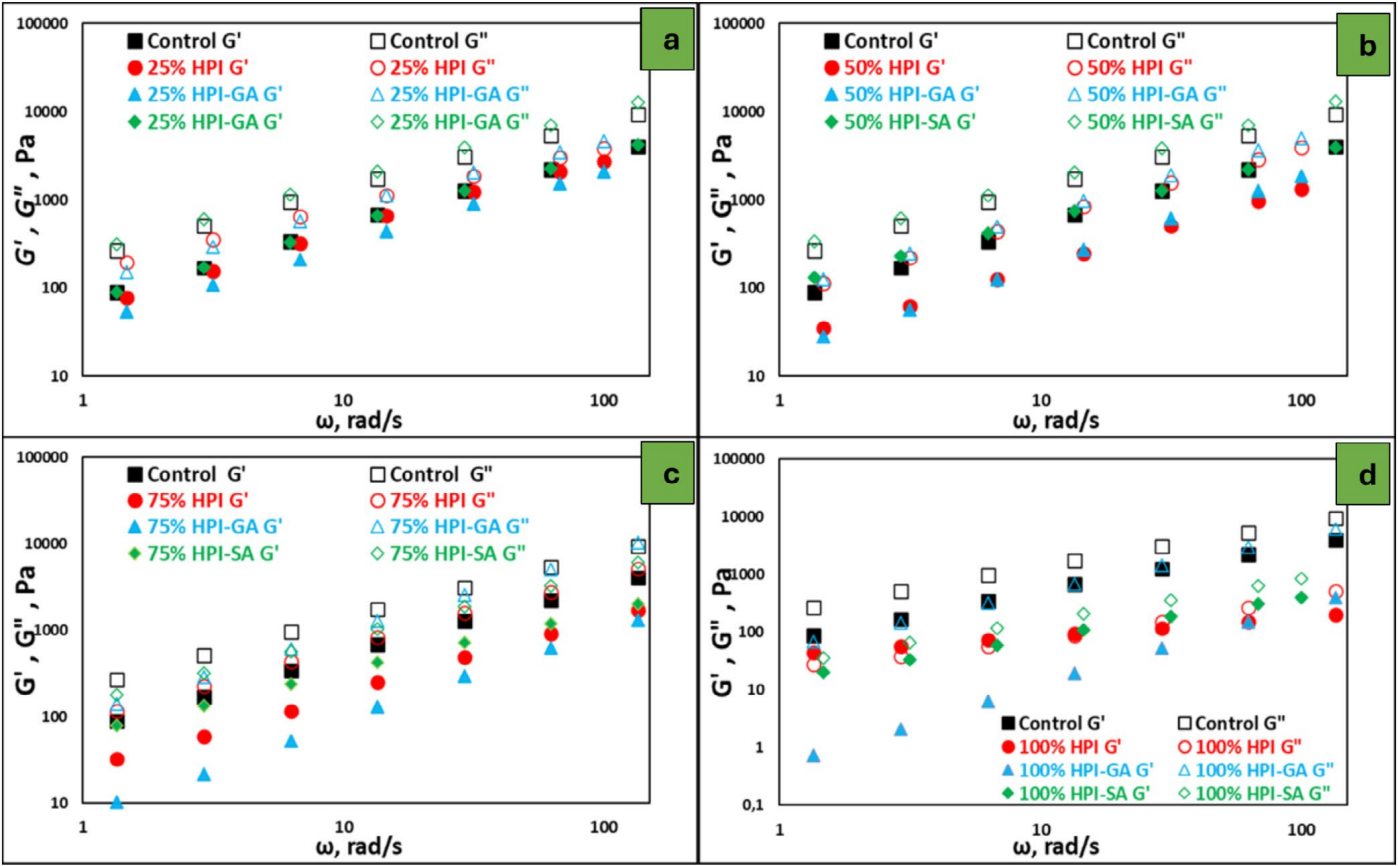
Dynamic rheological properties of nougat foam samples as a function of angular frequency (ω) at different egg white replacement levels: (a) 25%, (b) 50%, (c) 75%, and (d) 100%. The storage modulus (*G′*) and loss modulus (*G″*) of control and samples formulated with HPI, HPI–GA, and HPI–SA are presented. Filled symbols represent G′, while open symbols represent G″.

The viscoelastic behavior of the nougat foam samples was evaluated by frequency sweep tests, and the results are presented in Figure 3. For all formulations, both the storage modulus (G′) and the loss modulus (G″) increased with increasing angular frequency, indicating a frequency‐dependent response typical of structured, aerated sugar‐based systems. Throughout the examined frequency range, *G″* values were generally higher than *G′*, showing that the viscoelastic response of the nougat foams was mainly governed by viscous behavior (Liu et al. 2022). This indicates that the foams behaved as structured liquids with limited elastic character rather than as fully developed elastic networks. The control formulation followed this trend, which is consistent with its soft and highly aerated structure.

Replacing egg white powder with protein‐based alternatives influenced the magnitude of the viscoelastic moduli. Foams prepared with native HPI exhibited lower *G′* values, particularly at low frequencies, reflecting a weaker internal structure and reduced resistance to deformation under oscillatory shear. In comparison, foams containing glycosylated protein systems showed higher G′ values and a smaller difference between G′ and G″, especially at low and intermediate replacement levels (25% and 50%). This behavior suggests a partial reinforcement of the foam structure, even though viscous behavior remained dominant due to the improved viscoelastic properties. At higher replacement levels (75% and 100%), the dominance of G″ persisted, indicating that increasing protein or conjugate content did not result in a transition to elastic‐dominant behavior. Among the tested systems, foams containing HPI–SA consistently exhibited higher G′ values and a narrower separation between G′ and G″ than those containing HPI–GA or native HPI, pointing to a more structured and deformation‐resistant foam network.

The improved viscoelastic response of the conjugate‐containing foams can be attributed to protein glycosylation, which promotes stronger intermolecular interactions, greater hydration, and an increase in effective molecular volume. In particular, sodium alginate likely contributed to higher viscosity and better network connectivity through its strong water‐binding ability and interactions with the protein phase, leading to improved resistance to oscillatory deformation (Lazidis et al. 2016). Taken together, the dynamic rheological results indicate that, although viscous behavior dominated in all nougat foams, protein glycosylation, especially with sodium alginate, enhanced structural organization and viscoelastic strength. These findings are consistent with the trends observed in macroscopic appearance, apparent density, and steady‐shear rheology, and support the superior foam‐stabilizing performance of glycosylated hazelnut protein systems compared to native HPI.

### 3.3 Texture Analysis of Nougat Foams

#### 3.3.1 Spreadability

Spreadability results are presented in Table 1 in terms of firmness and work of shear. The control sample exhibited a relatively high firmness and work of shear, reflecting the strong structural resistance of the conventional egg white–based foam. Replacing egg white powder with native HPI generally reduced spreadability‐related resistance, particularly at high replacement levels. This effect was most pronounced for the 100% HPI formulation, which showed the lowest firmness and work of shear values, indicating a markedly weakened structure with minimal resistance to deformation during spreading.

In contrast, the conjugate‐containing systems showed a clear strengthening effect. This strengthening effect is consistent with previous studies reporting that protein–polysaccharide conjugation enhances interfacial film formation and promotes the development of a more cohesive and mechanically resistant network structure (Babu et al. 2024; X. Zhang, Wang, et al. 2024). At comparable replacement levels, HPI–SA produced the highest firmness and work of shear values, indicating the greatest resistance to spreading and a more cohesive structure. This effect peaked at 50% HPI–SA, which exhibited the maximum values for both parameters, suggesting an overly reinforced matrix under these conditions. HPI–GA samples also showed higher spreadability resistance than native HPI, but their values remained below those of the HPI–SA counterparts, indicating a more moderate structuring effect. Taken together, the spreadability data suggest that protein glycosylation improved the mechanical integrity of the foam matrix under shear deformation, with sodium alginate conjugation providing the strongest reinforcement among the tested systems.

#### 3.3.2 Back Extrusion

The back extrusion behavior of the nougat foam samples is summarized in Table 1 using peak force and positive area values, which reflect the resistance of the foam structure to compressive and extensional deformation. The control sample exhibited relatively high peak force and positive area values, indicating a firm and cohesive structure capable of resisting deformation during extrusion. Replacing egg white powder with native HPI led to a clear reduction in both parameters, particularly at high replacement levels. The 100% HPI foam showed the lowest peak force and positive area, revealing a weak structure with limited resistance to deformation and poor structural cohesion.

In contrast, foams containing glycosylated protein systems demonstrated markedly enhanced back extrusion resistance. Among all formulations, HPI–SA samples exhibited the highest peak force and positive area values, indicating the strongest resistance to extrusion and the most cohesive structure. This effect was especially pronounced at the 50% replacement level, where maximum values were observed, suggesting the formation of a highly reinforced foam network. HPI–GA samples also showed improved back extrusion behavior compared to native HPI, although their resistance remained lower than that of the HPI–SA counterparts. The differences between formulations suggest that protein glycosylation enhanced the ability of the foam matrix to withstand deformation, with sodium alginate conjugation providing the most effective reinforcement of the structural network under back extrusion conditions. These findings are in agreement with previous studies demonstrating that Maillard‐induced glycosylation improves the foaming capacity and stability of proteins by facilitating the formation of a denser and more viscoelastic interfacial layer. Furthermore, the extension of covalently linked polysaccharide chains into the aqueous phase enhances structural integrity and resistance to deformation through steric stabilization (S. Zhang, Liu, and Wu 2024).

### 3.4 Microstructural Imaging

**FIGURE 4:**
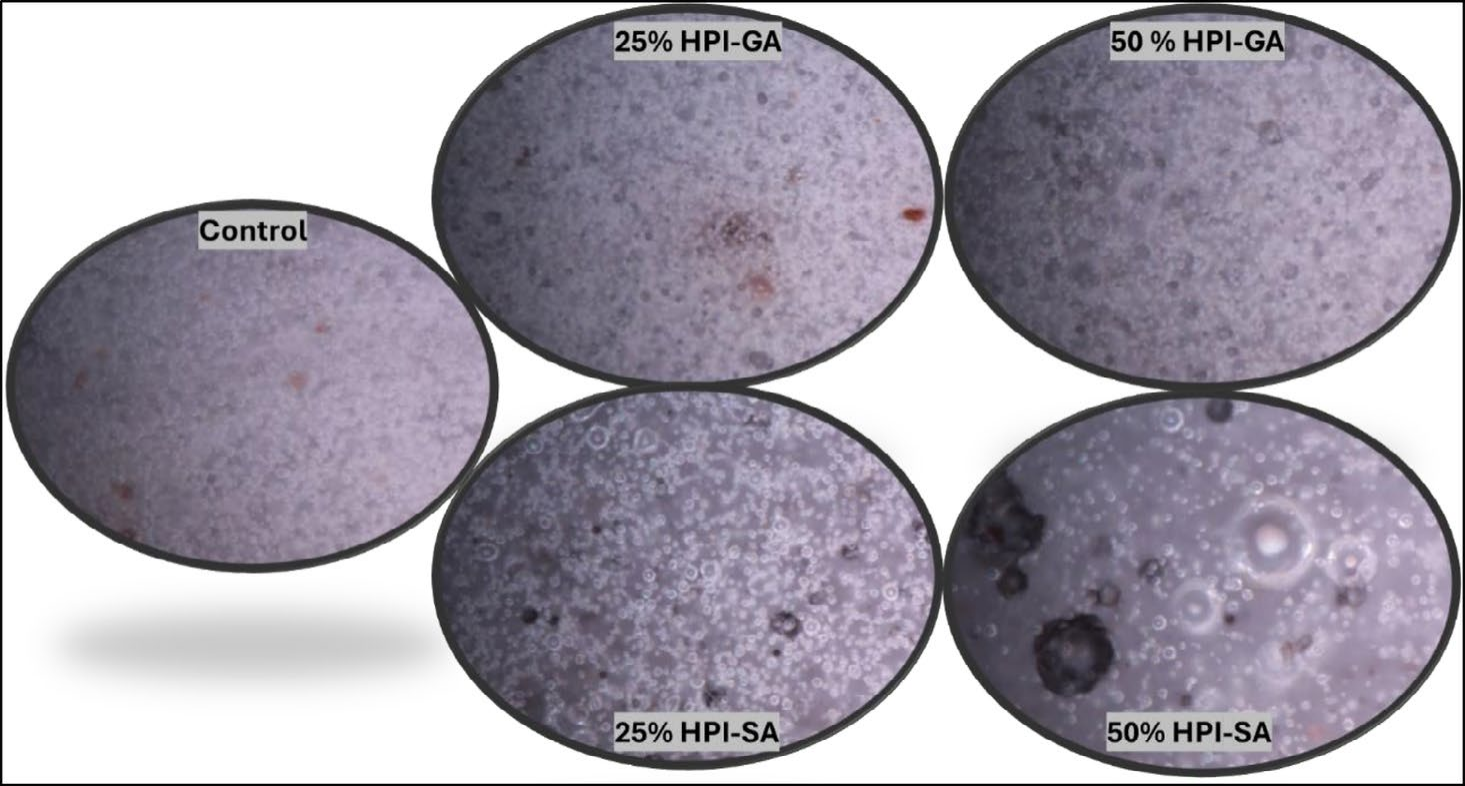
Microstructural images of nougat foam (NF) samples at different egg white replacement levels, including control, HPI–GA (25% and 50%), and HPI–SA (25% and 50%).

Representative optical micrographs of the control and conjugate‐containing nougat foam samples are presented in Figure 4. The control foam exhibited a relatively uniform microstructure with finely distributed air bubbles embedded within a continuous matrix, indicating efficient air incorporation and stable foam formation. Foams prepared with glycosylated hazelnut protein conjugates showed microstructural characteristics that varied depending on both the conjugate type and the replacement level. At 25% replacement, both HPI–GA and HPI–SA foams displayed a fine and relatively homogeneous distribution of air bubbles, comparable to that of the control sample. Although slightly larger air bubbles were observed in the HPI–SA foam at this level, they remained well dispersed and uniformly embedded within the continuous matrix. This observation suggests effective interfacial stabilization and indicates controlled air bubble growth rather than bubble coalescence (Dickinson 2010) which is consistent with the relatively low apparent density and stable rheological behavior observed at this level. At 50% replacement, more pronounced differences between the two conjugate systems became evident. HPI–GA foams exhibited a broader air bubble size distribution with the presence of localized larger bubbles, pointing to partial bubble growth and the onset of coalescence. This structural heterogeneity is in line with the reduced mechanical resistance and weaker viscoelastic response observed for HPI–GA samples. In contrast, HPI–SA foams maintained a more compact and interconnected microstructure, with air bubbles remaining well integrated within the surrounding matrix despite the presence of some larger bubbles. This behavior suggests that sodium alginate provided stronger interfacial and bulk‐phase support, enabling the foam structure to accommodate increased air incorporation without substantial destabilization, which is consistent with the higher storage modulus and improved textural resistance of these samples. These microstructural observations demonstrate that glycosylated hazelnut protein conjugates effectively stabilized air bubbles in nougat foams (Lazidis et al. 2016). Among the tested systems, HPI–SA exhibited superior structural integrity compared to HPI–GA, particularly at higher replacement levels. These findings are consistent with the enhanced mechanical resistance, viscoelastic behavior, and controlled apparent density observed for HPI–SA foams, confirming the strong relationship between microstructure and macroscopic foam properties.

These findings can also be interpreted within the broader context of structured food design, where controlling internal architecture plays a key role in determining product functionality. Recent advances in 3D food printing have demonstrated that formulation and processing conditions directly influence porosity, bubble distribution, and textural properties in protein‐based systems. In this regard, the ability of glycosylated hazelnut protein conjugates to modulate bubble structure and network organization highlights their potential for tailoring internal foam architecture, which is consistent with emerging approaches in designing structured and aerated food matrices (Lombardi, Consalvo, et al. 2025; Lombardi, Gala, et al. 2025).

### 3.5 FTIR

**FIGURE 5:**
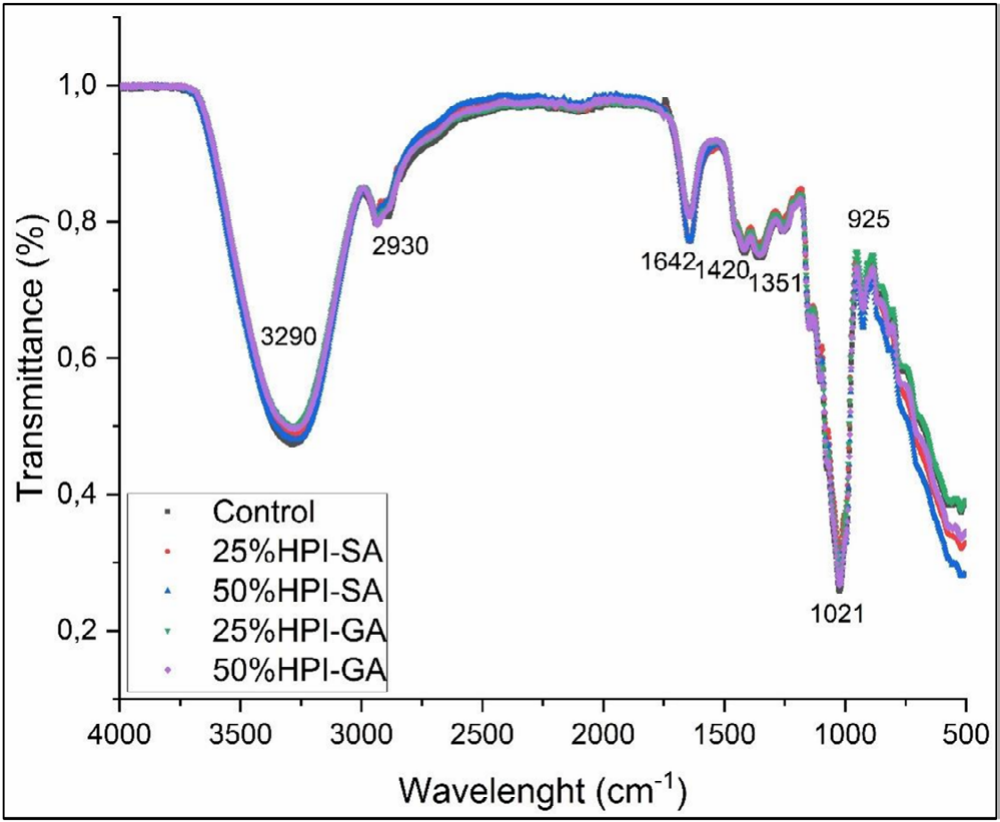
FTIR spectra of nougat foam (NF) samples at different egg white replacement levels, including control, HPI–SA (25% and 50%), and HPI–GA (25% and 50%).

The FTIR spectra of the control and conjugate‐containing nougat foam samples are shown in Figure 5. All samples exhibited broadly similar spectral profiles, indicating that foam preparation and partial replacement of egg white powder did not introduce new functional groups but rather affected the relative intensities and shapes of existing bands. The broad absorption band observed around 3290 cm^−1^ is attributed to O–H and N–H stretching vibrations, mainly associated with hydrogen‐bonded water, hydroxyl groups of polysaccharides, and amide A of proteins (Patel et al. 2025). Compared to the control sample, foams containing glycosylated protein conjugates, particularly HPI–SA, showed a slightly broader and more intense band in this region, suggesting enhanced hydrogen bonding and increased water binding capacity due to the presence of polysaccharide moieties. The weak band near 2930 cm^−1^ corresponds to C–H stretching vibrations of aliphatic –CH_2_ and –CH_3_ groups, originating from protein side chains and minor lipid components. No pronounced differences were observed among samples in this region, indicating that hydrophobic aliphatic structures were not significantly altered by conjugation or replacement level. In the fingerprint region, the band at approximately 1642 cm^−1^ is mainly assigned to the amide I vibration (C = O stretching) of proteins, with possible contributions from absorbed water (Hoseini‐Yazdi et al. 2025). The band position remained essentially unchanged among samples, suggesting no major shift in the dominant protein secondary‐structure features. However, the 50% HPI–SA sample exhibited a slightly higher band intensity in this region, which may indicate stronger hydrogen bonding and/or a higher contribution of bound water and protein–polysaccharide interactions around the protein backbone, consistent with the more structured foam matrix formed in the presence of sodium alginate. The absorption bands around 1420 and 1351 cm^−1^ are associated with C–H bending and COO^−^ symmetric stretching vibrations, which can originate from both protein side chains and polysaccharide components. These bands were more pronounced in the spectra of HPI–GA and HPI–SA foams, supporting the contribution of polysaccharide groups within the foam matrix. The strong band observed at approximately 1021 cm^−1^, together with the shoulder near 925 cm^−1^, is characteristic of C–O–C and C–O stretching vibrations of polysaccharides (Alarape et al. 2024). The increased intensity of these bands in conjugate‐containing samples, particularly for HPI–SA formulations, confirms the presence and structural contribution of gum arabic and sodium alginate within the foam network.

These spectral features suggest that conjugation‐induced hydrogen bonding and increased polysaccharide incorporation enhanced the hydration and structural cohesion of the interfacial protein layer. This improved interfacial organization likely contributed to the formation of a more viscoelastic and resistant network, which is consistent with the higher storage modulus, improved textural resistance, and lower apparent density observed in conjugate‐containing samples. Therefore, the FTIR results provide molecular‐level support for the enhanced foam stability and structural integrity of glycosylated hazelnut protein systems.

## 4 Conclusions

This study demonstrated that glycosylated hazelnut protein conjugates can act as effective functional alternatives to egg white proteins during the foam formation stage of nougat processing. Replacement of egg white powder with native hazelnut protein isolate resulted in weaker foam structures, as reflected by higher apparent density, reduced mechanical resistance, and lower rheological strength. In contrast, Maillard‐type conjugation with gum arabic or sodium alginate markedly enhanced the foam‐stabilizing performance of hazelnut protein, as evidenced by improved macroscopic appearance, controlled apparent density, stronger textural resistance, and enhanced steady‐shear and dynamic viscoelastic behavior. Among the tested systems, HPI–SA conjugates consistently outperformed HPI–GA, particularly at 25% and 50% replacement levels. These formulations exhibited foam properties closest to those of the egg white control, combining sufficient aeration with improved structural integrity, deformation resistance, and microstructural stability. This improved performance was supported by microstructural observations and FTIR analysis, indicating stronger protein–polysaccharide interactions, increased water binding, and a more cohesive foam network. Based on these findings, replacement levels of 25% and 50% were identified as the most suitable for maintaining balanced foam structure and functionality. These levels will be further investigated in future studies involving more complex systems, particularly emulsion‐type nougat filling formulations, where interactions between foam and fat–cocoa phases are expected to play a critical role. From an industrial perspective, hazelnut protein represents a promising plant‐based alternative to egg white proteins due to its sustainability, local availability, and compatibility with clean‐label formulations. The improved functional performance achieved through protein–polysaccharide conjugation further supports its applicability in aerated confectionery systems, highlighting its potential as a cost‐effective and industrially adaptable ingredient. While the present study focuses on the intermediate foam stage, future work should evaluate the performance of these systems within the final nougat product to better assess their applicability in real processing conditions.

## Author Contributions

**Nevzat Konar:** writing – review and editing, investigation, supervision, conceptualization. **Sultan Demirci:** methodology, formal analysis. **Ilyas Atalar:** writing – original draft, investigation, formal analysis, supervision, conceptualization, project administration. **Ceren Elmaci:** methodology, formal analysis. **Serdar Marasli:** methodology, formal analysis. **Abdullah Kurt:** conceptualization, investigation, writing – original draft, formal analysis.

## Funding

This work was supported by Türkiye Bilimsel ve Teknolojik Araştırma Kurumu, 123O911.

## Disclosure

All authors have read and approved the final version of the manuscript. The corresponding author had full access to all of the data in this study and takes complete responsibility for the integrity of the data and the accuracy of the data analysis.

## Ethics Statement

This study does not involve any human or animal testing.

## Conflicts of Interest

The authors declare no conflicts of interest.

## Data Availability

The data that support the findings of this study are available from the corresponding author upon reasonable request.
